# Clearance of transaminases during normothermic *ex situ* liver perfusion

**DOI:** 10.1371/journal.pone.0215619

**Published:** 2019-04-24

**Authors:** Mariusz Bral, Nader Aboelnazar, Sanaz Hatami, Aducio Thiesen, David L. Bigam, Darren H. Freed, A. M. James Shapiro

**Affiliations:** 1 Department of Surgery, University of Alberta, Edmonton, Canada; 2 Department of Pathology, University of Alberta, Edmonton, Canada; University of Colorado, Anschutz Medical Campus, UNITED STATES

## Abstract

**Background:**

One of the most promising applications of liver normothermic machine perfusion (NMP) is the potential to directly assess graft viability and injury. In most NMP studies, perfusate transaminases are utilized as markers of graft injury. Our aim was to further elucidate the metabolism of transaminases by healthy porcine livers during NMP, specifically whether such livers could clear circuit perfusate transaminases.

**Methods:**

A highly concentrated transaminase solution was prepared from homogenized liver, with an aspartate aminotransferase (AST) level of 107,427 U/L. Three livers in the treatment group were compared to three controls, during 48 hours of NMP. In the treatment group, the circuit perfusate was injected with the transaminase solution to artificially raise the AST level to a target of 7,500 U/L. Perfusate samples were taken at two-hour intervals and analyzed for biochemistry until NMP end. Graft oxygen consumption and vascular parameters were monitored.

**Results:**

Compared to controls, treated perfusions demonstrated abrupt elevations in transaminase levels (p>0.0001) and lactate dehydrogenase (LDH) (p>0.0001), which decreased over time, but never to control baseline. Liver function, as demonstrated by lactate clearance and oxygen consumption was not different between groups. The treatment group demonstrated a higher portal vein resistance (p = 0.0003), however hepatic artery resistance was similar. Treated livers had higher bile production overall (p<0.0001).

**Conclusions:**

Addition of high levels of transaminases and LDH to a healthy porcine liver during ex situ perfusion results in progressive clearance of these enzymes, suggesting preserved liver metabolism. Such tolerance tests may provide valuable indicators of prospective graft function.

## Introduction

Due to a worldwide shortage of organs available for transplantation, there is increasing pressure to consider more marginal and ‘extended criteria’ liver grafts, in hopes of expanding the donor pool. It is well recognized that such grafts are not optimally preserved with current static cold storage (SCS), resulting in increased instances of both short and long term post-transplant complications. It is increasingly important that such grafts undergo some form of functional assessment before transplant to minimize any adverse outcomes.

Normothermic machine perfusion (NMP) has shown potential in resuscitating marginal liver grafts, and can improve the quality and quantity of transplanted livers [1]. Further, one the most promising applications of NMP is the possibility of dynamic, ‘real-time’ graft viability and injury assessment which optimally occurs under normothermic conditions to assess the functional capacity of an organ with physiologic metabolism. Many surrogate markers of graft viability and injury have been utilized, however none have been validated in the clinical setting. The ideal biomarker would be specific, easily processed, inexpensive, with a quick ‘turn around’ time. Almost ubiquitously across all clinical and experimental liver *ex situ* studies, perfusate transaminases are used as markers to inform of hepatocellular injury **(Table 1)**. Indeed, perfusate transaminases have been shown to correlate with post-transplant graft transaminase levels, and graft and recipient survival [2, 3].

**Table 1:** Selected normothermic ex situ liver perfusion studies utilizing transaminases for ‘on circuit’ graft injury assessment.

**Study**	**Year**	**Study Type/Device**	**Species**	**Sample Size (group)**	**Perfusate Assessment**	**Outcome Assessment**	**NMP Transaminase Correlation to Post-transplant Transaminase**
Watson *et al*. [4]	2018	Clinical Transplant (Liver Assist)	Human	47	In graph form.	41/46 livers had ALT <6000 I/U at 2 hours of perfusion. One liver with ALT 9490 I/U resulted in PNF.	Yes. ALT after 2 hours of perfusion with peak ALT (days 1–7) post-transplant (R = 0.73, p = 0.0001)
Nasralla *et al*. [1]	2018	Clinical Transplant (OrganOx *metra*)	Human	220	Not reported.	Peak AST 488.1 (U/L) NMP vs 964.9 (U/L) SCS (days 1–7).	Not reported
Watson *et al*. [5]	2017	Clinical Transplant (Liver Assist)	Human	12	In graph form.	In graph form.	Yes. ALT after 2 hours of perfusion with peak ALT (days 1–7) post-transplant (R = 0.56, p = 0.005)
Mergental *et al*. [6]	2016	Clinical Transplant (OrganOx *metra* and Liver Assist)	Human	5	Not reported.	In graph form.	
Bral *et al*. [7]	2016	Clinical Transplant (OrganOx *metra*)	Human	9	DCD livers much higher AST when compared to DBD (p < 0.001).	No difference in peak AST (day 1–7) levels between NMP and SCS grafts (p = 0.24).	Not Reported
Selzner *et al*. [8]	2016	Clinical Transplant (OrganOx *metra*)	Human	10	Peak AST 1647 (U/L), peak ALT 444 (U/L).	NMP livers had lower peak AST and ALT (day 1–3) compared to SCS (not significant).	Not reported
Ravikumar *et al*. [9]	2016	Clinical Transplant (OrganOx *metra*)	Human	20	Not reported.	Peak AST 417 (U/L) NMP vs 902 (U/L) SCS (days 1–7) (p = 0.34).	Not reported
Watson *et al*. [10]	2016	Clinical Transplant (Liver Assist)	Human	1	In graph form.	Peak ALT (days 1–7) 1198 (IU/L).	Not reported
Perera *et al*. [11]	2016	Clinical Transplant (Liver Assist)	Human	1	Not reported.	Peak ALT (days 1–7) 1215 (IU/L).	Not reported
Vogel *et al*. [12]	2017	Experimental Perfusion	Porcine	4	End perfusion (48 hrs) ALT 52.5 +/- 38.1 U/l	Day 5 ALT was 31.0 +/- 1.4 U/l.	Not reported
Vogel *et al*. [13]	2016	Experimental Perfusion	Human	13	In graph form.	Not transplanted.	Not applicable
Banan *et al*. [14]	2015	Experimental Perfusion	Porcine	12	NMP preserved DCD grafts had lower AST and ALT compared to SCS (p<0.01).	Not transplanted.	Not applicable
Nassar *et al*. [15]	2014	Experimental Perfusion	Porcine	20	NMP preserved livers had lower AST (p = 0.002) and ALT (p = 0.009) compared to SCS (p<0.01).	Not transplanted.	Not applicable
Op den Dries *et al*. [16]	2013	Experimental Perfusion	Human	4	In graph form. ALT stable throughout perfusion.	Not transplanted.	Not applicable
Boehnert *et al*. [17]	2013	Experimental Transplant	Porcine	6	NMP preserved grafts had six- fold lower ALT compared to SCS (p <0.001).	NMP preserved DCD grafts had lower mean AST compared to SCS.	Not reported
Xu *et al*. [18]	2011	Experimental Perfusion	Porcine	6	Livers with 60 min of warm ischemia had higher perfusate ALT levels (p< 0.05).	Not transplanted.	Not applicable
Fondevila *et al*. [19]	2011	Experimental Transplant	Porcine	6	NECMO perfused livers had lower AST 94 (38–148) compared to NMP livers 213 (119–413) U/L.	In graph form.	Not reported
Brockmann *et al*. [3]	2009	Experimental Transplant	Porcine	5	Significantly lower AST and ALT in livers preserved with NMP vs SCS (DCD model).	In graph form.	Yes. Successful livers AST 964+/-302 and ALT 62 +/- 10 vs AST 3198 +/-677, ALT 223+/-75 at 4 hours from NMP start
Reddy *et al*.[20]	2004	Experimental Perfusion	Porcine	5	Circuit transaminases lower in immediate NMP group compared to NMP + 4 hrs SCS	Not transplanted.	Not applicable.
Butler *et al*. [21]	2002	Experimental Perfusion	Porcine	5	ALT level 51.4 (U/L) after 72 hours of NMP.	Not transplanted.	Not applicable.
Schoen *et al*. [22]	2001	Experimental Transplant	Porcine	6	Not reported.	AST levels lower in livers preserved with NMP.	Not reported.

Aspartate aminotransferase (AST), Alanine aminotransferase (ALT), Donation after brain death (DBD), Donation after cardiac death (DCD), Normothermic machine perfusion (NMP), Normothermic extra-corporeal membrane oxygenation (ECMO), Primary non-function (PNF), Static cold storage (SCS).

As such, transaminases are often used in combination with graft lactate clearance and bile production during NMP to determine the eligibility of a particular graft for implantation.

Herein, we aim to further elucidate the metabolism of transaminases by healthy porcine livers during NMP, specifically whether healthy livers could clear transaminases from a NMP perfusate. Such information would potentially further shed light on whether transaminases could be used as a functional test to assess liver viability.

## Methods

### Study design overview

**Fig 1:**
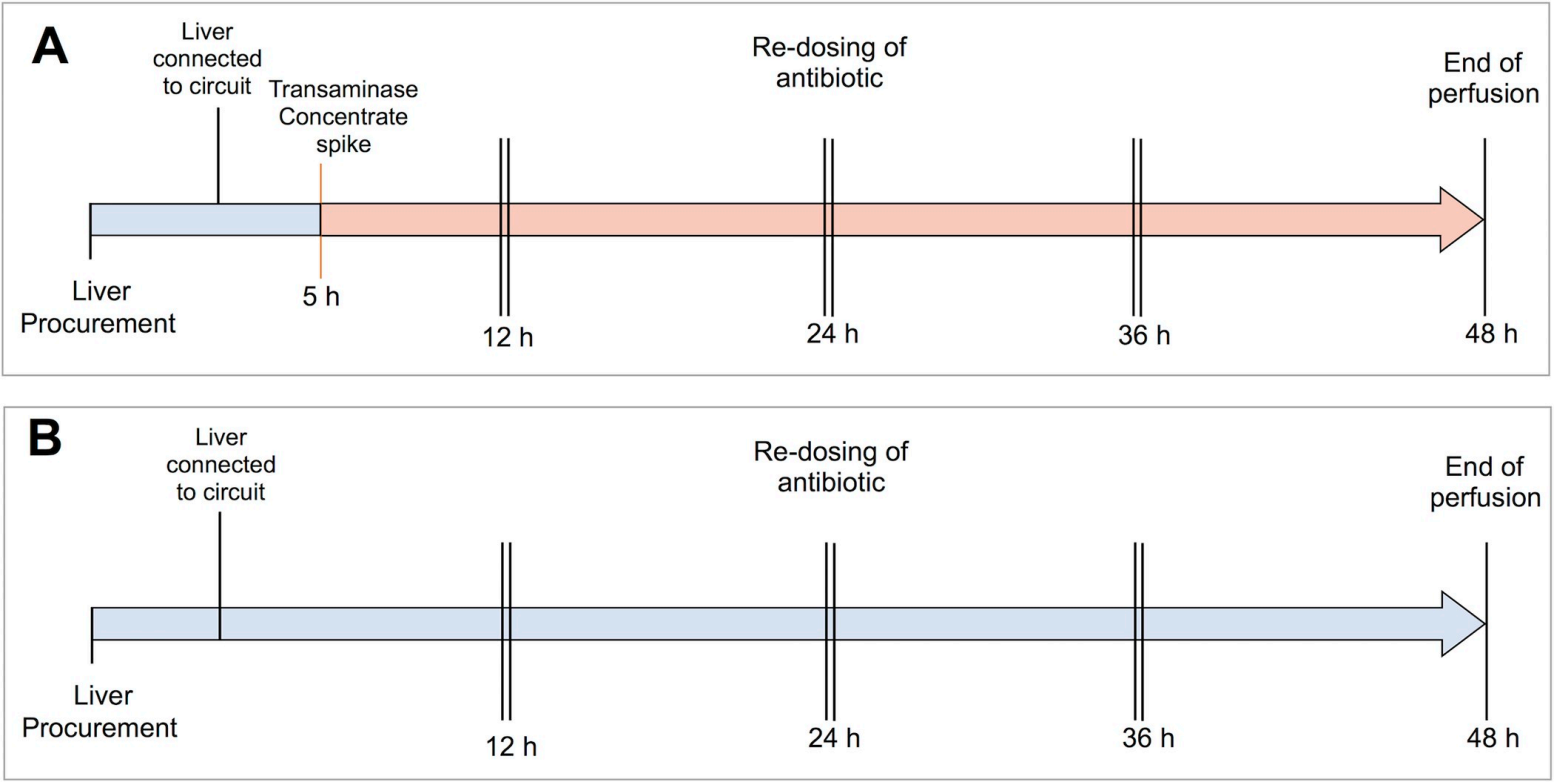
Flow diagrams of the experimental design. (A) Schematic diagram of the ex situ liver perfusion treatment group, perfused for 48 hours. (B) Schematic diagram of control livers perfused for 48 hours.

The Institutional Animal Care Committee at the University of Alberta approved the experimental protocol. A total of 7 Landrance pigs were used for these experiments. Three livers were allocated to the treatment group, and perfused on our locally designed *ex situ* circuit under normothermic conditions for 48 hours, and compared to three control livers perfused for the same duration. In the treatment group, after stable perfusion was established for 5 hours, the circuit perfusate was injected with a prepared high concentration transaminase solution to artificially elevate the circuit AST to a projected target level of 7,500 U/L **(Fig 1)**. Throughout all perfusions, perfusate samples were drawn at two-hour intervals, and frozen at -80°C until biochemical analysis could be performed. Samples were analyzed for AST, alanine aminotransferase (ALT), LDH, and lactate. Blood gas analysis was also performed at two-hour intervals, and graft oxygen consumption and vessel resistance was monitored.

### Donor liver procurement

Livers were procured from 35–45 Kg domestic pigs and connected to our experimental *ex situ* circuit. Donor pigs were premedicated with Atropine (0.05 mg/Kg) (Rafter 8, Calgary, Canada) and Ketamine (20 mg/Kg) (Bimeda, Cambridge, Canada), orotracheal intubation was performed, and general anesthesia was sustained with 2% isofluorane (Fresenius Kabi Canada Ltd, Richmond Hill, Canada). Through a midline laparotomy livers were retrieved as previously described [21, 23]. All livers were dissected and vascular elements appropriately isolated. Through a median sternotomy, a two stage venous cannula was inserted into the right atrium. Intravenous heparin (30,000 (Fresenius Kabi Canada Ltd, Richmond Hill, Canada), was administered, and the infra-renal aorta was cannulated with a 20 French cannula in preparation for cold flush. Exsanguination of the animals was then performed using the right atrial cannula, and the collected blood was used to prime the perfusion circuit. The aorta was cross-clamped, the suprahepatic vena cava was divided in the chest for venous venting, and the abdominal viscera were flushed with 2 liters of cold (4°C) Histidine-Tryptophan-Ketoglutarate (Custodiol HTK, Methapharm Inc., Brantford, ON, Canada).

### Preparation of liver transaminase concentrate

A modified isolation protocol was used to prepare a highly concentrated transaminase solution. A single porcine liver (917 g) was mechanically homogenized, following which the liquefied tissue was frozen at -80°C. This tissue homogenate was then thawed, and aliquoted into 30 mL samples and then subjected to a further 2 minutes of homogenization using a PowerGen125 (Thermo Fisher Scientific, NY). Samples were kept on ice at all times. The liquefied liver samples were then sonicated using a Virtis Virsonic 100 Ultrasonic Cell Disrupter (VirTis, NY), two times, for a duration of 30 seconds each. Samples were then centrifuged at 1,600 X G for 15 min (Sorvall Legend XTR, Thermo Fisher Scientific, MA). The supernatant was collected, and refrozen in 50 mL aliquots at -80°C. A sample of this was processed for AST levels. The sample was then refrozen at -80°C, thawed again, and resent to the lab to ensure enzyme stability. The reported AST concentration was reported as 107,427 U/L. This concentrated transaminase supernatant was used as an additive in the treatment group to raise the AST level to a target of 7,500 U/L. For comparison, tissues from alternative solid abdominal organs including kidney and spleen were also processed in this fashion and analyzed for biochemistry, but were ultimately not applied to any *ex situ* perfusions, as these latter solutions were also found to be high in transaminases.

### *Ex situ* perfusion circuit design

**Fig 2:**
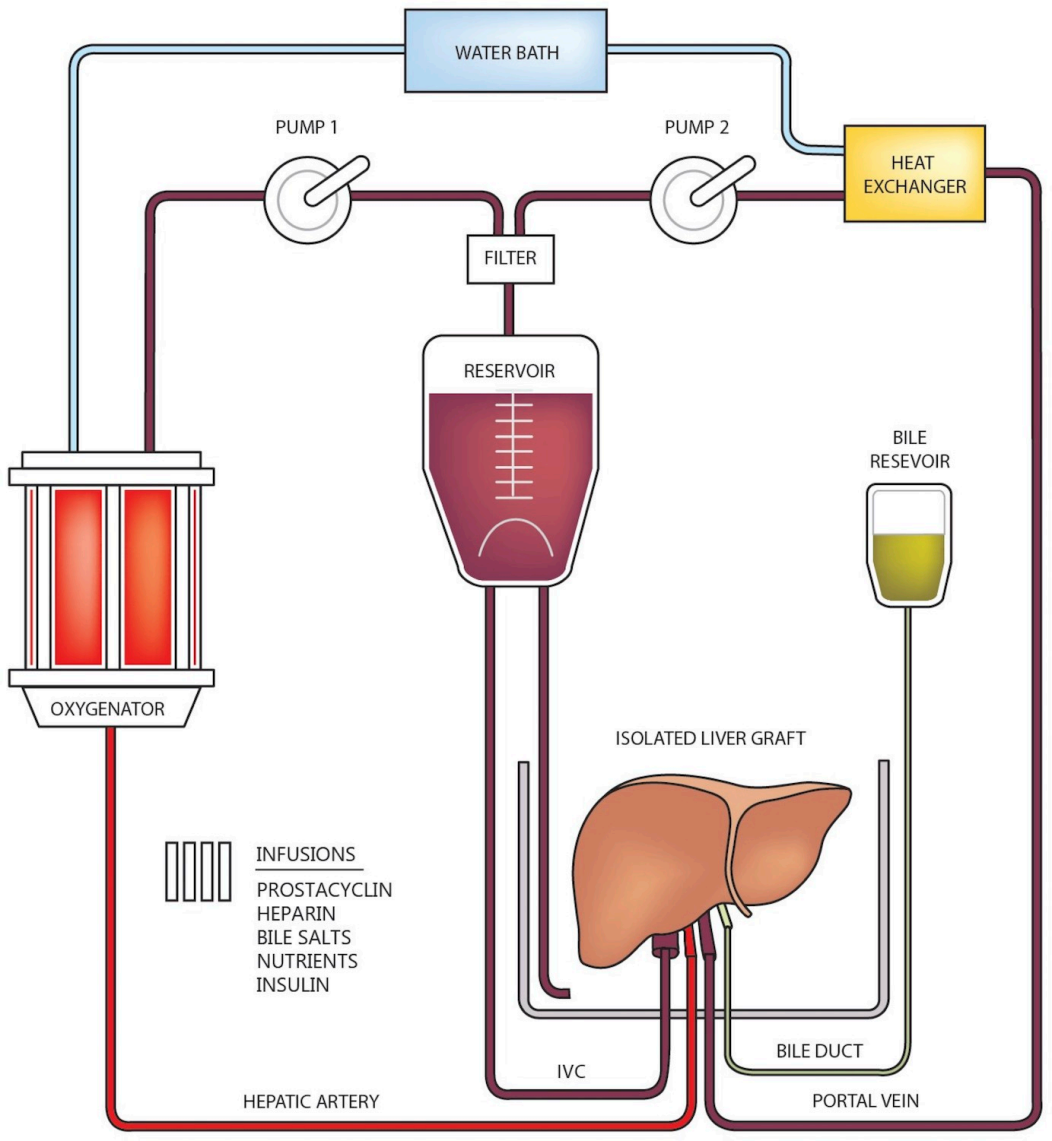
Schematic diagram of the experimental ex situ circuit. In this study prostacyclin, bile salts and nutrients were omitted from the infusions. Reprinted from ‘Mariusz Bral, Boris Gala-Lopez, Aducio Thiesen, Sanaz Hatami, David L. Bigam, Darren M. Freed, AM James Shapiro, Determination of Minimal Hemoglobin Level Necessary for Normothermic Porcine Ex Situ Liver Perfusion, *Transplantation*. 2018;102(8):1284–1292, with permission from Transplantation.

The locally-designed experimental *ex situ* perfusion circuit was assembled using the following components: a Medtronic Affinity NT oxygenator, two BPX-80 Bi-Medicus centrifugal pumps (Medtronic, Minneapolis, MN), and a leukocyte arterial blood filter (LeukoGuard LG, PALL Medical, Port Washington, NY) **(Fig 2)**. The centrifugal pumps were computer controlled to maintain the desired hepatic artery and portal vein pressures. Oxygen and carbon dioxide flows were titrated through the membrane oxygenator to maintain a partial pressure of arterial oxygen between 130 and 200 mmHg, and a partial pressure of carbon dioxide of 35 to 45 mmHg.

A volume of 1000 mL of whole blood was added to 750 mL of Krebs-Henseleit with albumin solution (Glucose, sodium chloride, potassium chloride, calcium chloride, magnesium chloride, sodium bicarbonate, sodium phosphate monobasic, 8% bovine serum albumin). The circuit was primed with bolus additives including calcium gluconate 10 mls 10% solution (Fresenius Kabi, Richmond Hill, Canada), cefuroxime 750mg (SteriMax Inc., Oakville, Canada), methylprednisolone sodium succinate 500 mg (Pfizer Canada Inc., Kingston, Canada), and sodium heparin 10,000 U (Pharmaceutical Partners Canada, Richmond Hill, Canada). Sodium bicarbonate (8.4%) was added (Hospira, Montreal, Canada) as needed to maintain pH between 7.35–7.45. Cefuroxime 750mg (SteriMax Inc., Oakville, Canada) and was re-bolused at 24 hours of perfusion. Continuous infusions were established of 2 IU/hour of regular insulin (Eli Lilly Canada Inc., Toronto, Canada), and 1,000 units/hour of sodium heparin.

### *Ex situ* liver perfusion

Following procurement, livers were flushed with 1 L of 0.9% normal saline. Livers were connected to our *ex situ* perfusion circuit at a temperature of 39°C, the normal pig core body temperature. Livers were perfused through both the hepatic artery and portal vein. Both of the perfused vessels were under automated computer pressure control, with the hepatic artery perfused at a target of 70 mmHg and the portal vein perfused at a target pressure of 2 mmHg. All perfusions were performed for durations of 48 hours.

In the designated treatment group (n = 3), perfusions were allowed to proceed for 5 hours, at which point the circuit was injected through the portal vein component with a calculated volume of the prepared high concentration transaminase supernatant to raise the AST level up to a target of 7,500 U/L. Following this intervention, liver perfusion was continued to 48 hours. In the control liver group (n = 3), perfusions were established and allowed to proceed for 48 hours, without any intervention. At 2 hour intervals, perfusate samples were withdrawn, centrifuged, and the supernatant was stored at minus 80°C until biochemical analysis was performed. Blood gasses were also analyzed every 2 hours. Tissue samples were taken at the end of each perfusion for blinded histological analysis.

### Perfusate composition analysis

Hemoglobin, electrolyte, pH, lactate and partial pressures of oxygen and carbon dioxide were measured using the ABL Flex Analyzer (Radiometer Medical ApS, Bronshoj, Denmark). Perfusate samples were obtained from the hepatic artery circuit and analyzed for levels of AST, ALT, LDH using a Beckman Coulter Unicel Dxc800 Syncron (Brea, California, USA), in our hospital clinical laboratory.

### Hepatic oxygen consumption and vascular resistance

Liver graft oxygen consumption was calculated using the Fick equation and compared at two-hour intervals. Hepatic artery and portal vein vascular resistance was calculated by dividing the vessel pressure by the flow indexed to 100 grams of liver tissue.

### Histology

Liver tissue samples were obtained at the end of each perfusion, and were fixed in 10% formalin. Tissue was embedded in paraffin, stained with hematoxylin and eosin, and examined in a blinded fashion by an independent expert pathologist, blinded to the experimental group, who assigned a semi-quantitative score to evaluate for hepatocyte injury and bile sequestration. Biopsy tissue was examined for necrosis (0- absent, 1- pericentral, 2- Zone 2 and 3, 3- panlobular); hemorrhage (score 0- absent, 1- focal, 2- zonal, 3- panlobular), cholestasis (score 0- absent, 1- present); and sinusoidal dilatation (score 0- none, 1- mild, 2- moderate, 3- severe), as previously reported [3].

### Statistical analysis

Data are represented as means ± standard error of the means (SEM). Differences between continuous variables were compared using a repeated measures ANOVA or the Mann Whitney U-test. Overall comparison between groups was performed with a 95% confidence interval. A p-value of <0.05 was considered statistically significant and all the analysis was performed using Graphpad Prism (GraphPad Software Inc., La Jolla, CA, USA).

## Results

The *ex situ* circuit was primed concomitantly with donor procurement surgery and NMP was established in all cases without difficulty. Mean starting hemoglobin concentration of the perfusates was 62.8 ± 8.7 g/L. There were no technical complications during the machine perfusions for any of the livers. The starting enzyme values in the prepared concentrated supernatant were as follows: AST 107,427 U/L, ALT 2,788 U/L and LDH 28,050 U/L. Tissue concentrate biochemistry from other solid abdominal organs, not used in any perfusions, was as follows: Kidney- AST 44,383 U/L, ALT 2,128 U/L, LDH 23,995 U/L, Lac 6.41 mmol/L; Spleen- AST 9,004 U/L, ALT 273 U/L, LDH 26,041 U/L, Lac 10.57 mmol/L.

**Fig 3:**
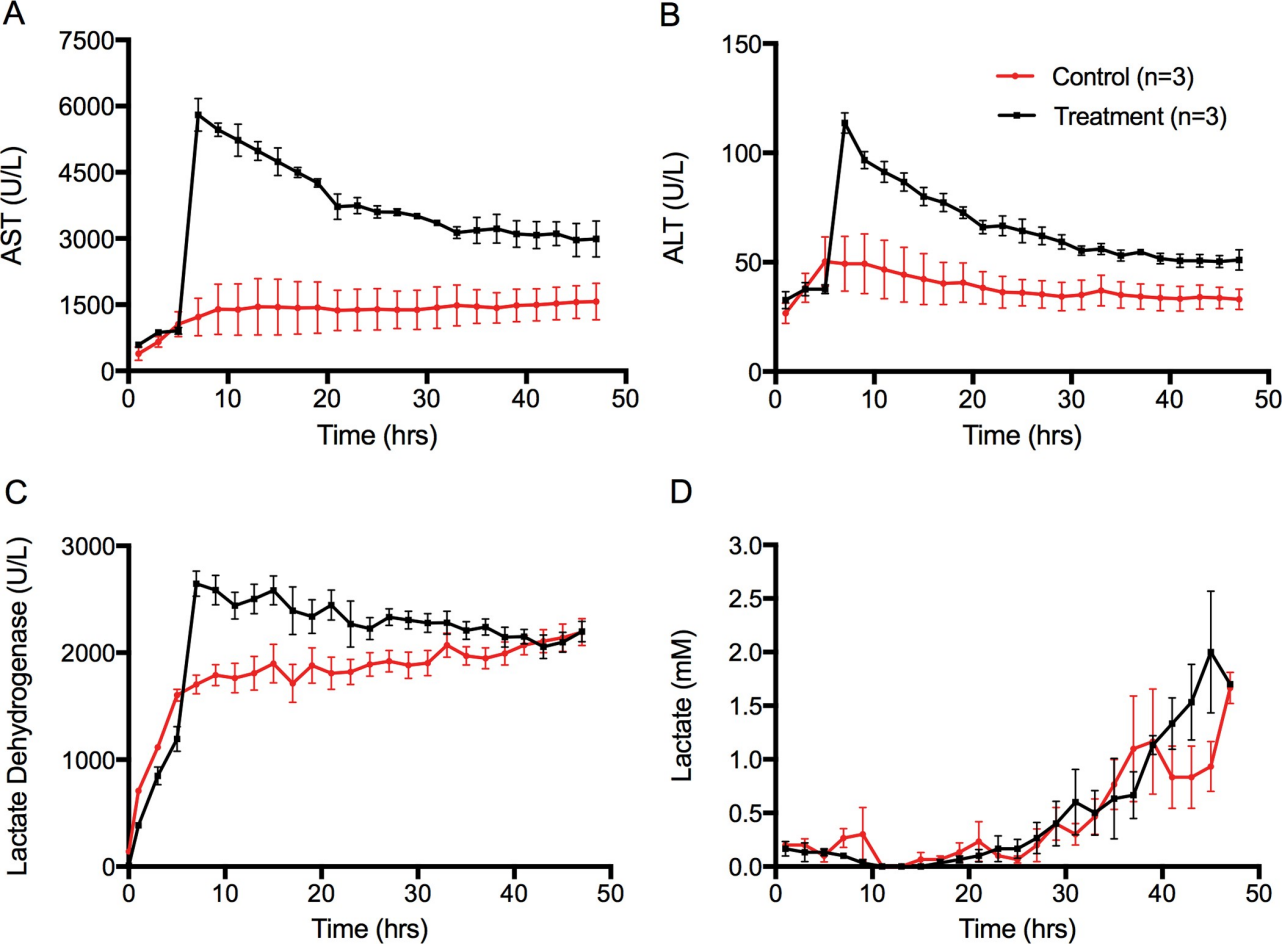
‘On circuit’ perfusate biochemistry during NMP. (A) Circulating AST perfusate levels during NMP, (p<0.0001). (B) Circulating ALT perfusate levels (p<0.0001). (C) Ex situ circulating perfusate lactate dehydrogenase levels at each hemoglobin level (p<0.0001). (D) Lactate levels at each time point (p = 0.24). Data points show means and SEM, 95% confidence interval. Aspartate aminotransferase (AST), Alanine aminotransferase (ALT).

Transaminase levels in the re-circulating ex situ perfusate initially began to increase in all cases. Perfusate transaminase levels sharply elevated in the treatment group at the intervention time point (5 hours), and were significantly higher for both AST (p<0.0001) and ALT (p<0.0001) **(Fig 3A and 3B).** The transaminase levels decreased over the duration of perfusion in the treatment group, but never to the same level as in the control group. In the control group, transaminase levels initially increased and then stabilized out after hour 8 of perfusion. LDH levels also increased sharply at the intervention point in the treatment group (p<0.0001) **(Fig 3C)** and also decreased over time, to the level of the controls. In the control group, LDH increased, and then leveled at approximately perfusion hour 8. Lactate levels increased in all perfusions, with no statistical significance between groups (p = 0.12) **(Fig 3D)**.

**Fig 4:**
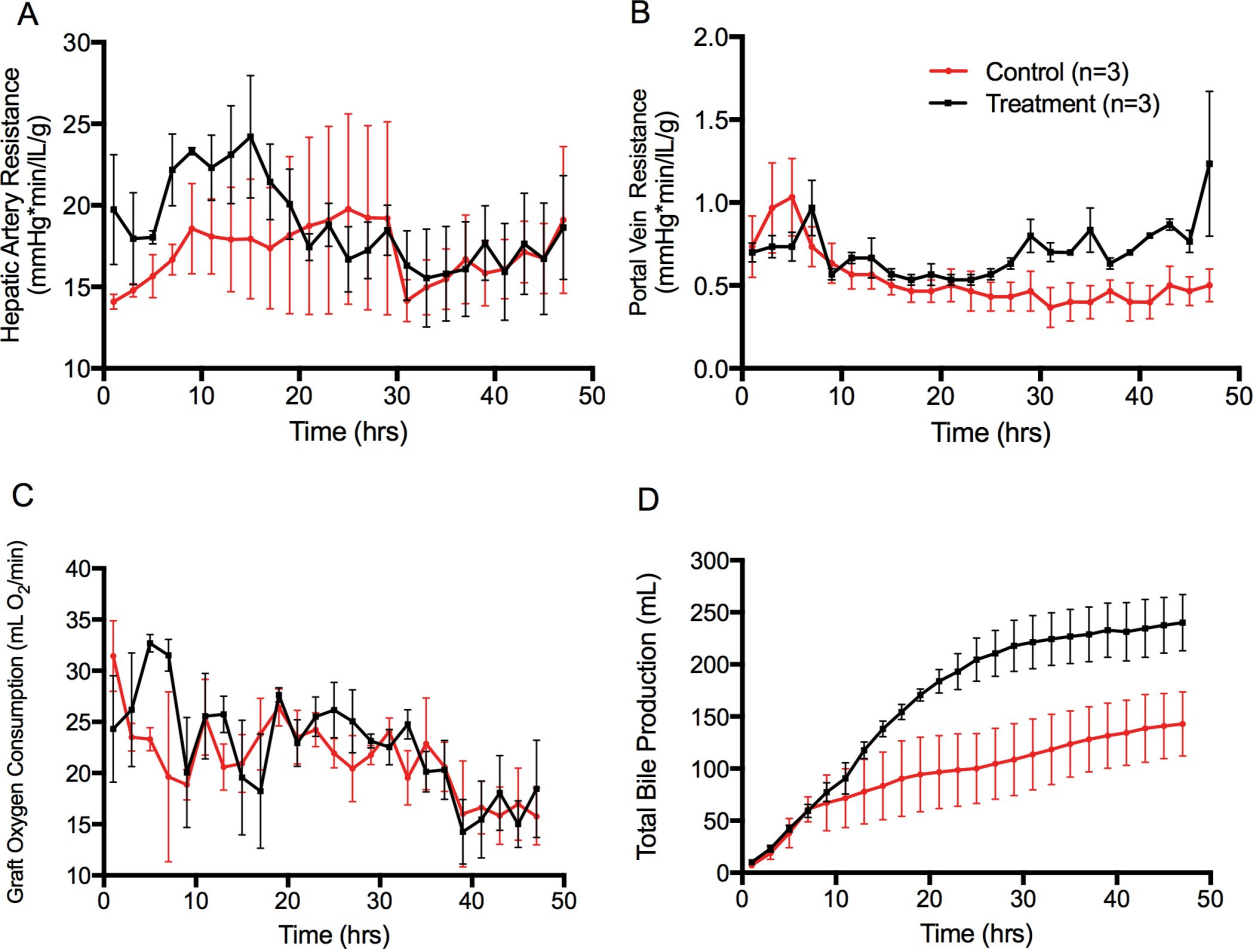
Vascular parameters and bile production during NMP. (A) Hepatic oxygen consumption during NMP (p = 0.41). (B) Total bile output during NMP (p<0.0001). (C) Hepatic artery resistance during NMP (p = 0.14). (D) Portal vein resistance during NMP (p = 0.0003). Data points show means and SEM, 95% confidence interval. Normothermic machine perfusion (NMP).

Hepatic vascular parameters demonstrated changes between groups. Hepatic artery resistance (HAR) increased in the treatment group at the time of the intervention, but then decreased to the baseline of the controls with no statistical difference (p = 0.14) **(Fig 4A)**. Portal vein resistance (PVR), also increased at the time of the intervention, returned to control levels, and then demonstrated a steady rise until the end of perfusion duration (p = 0.0003) **(Fig 4B)**. Liver graft oxygen consumption fluctuated over the duration of perfusions, but was not statistically different between groups, decreasing over the perfusion duration (p = 0.41) **(Fig 4C).** Total bile output in both groups steadily accumulated in both groups, but was statistically greater in the intervention group (p<0.0001) **(Fig 4D).**

**Fig 5:**
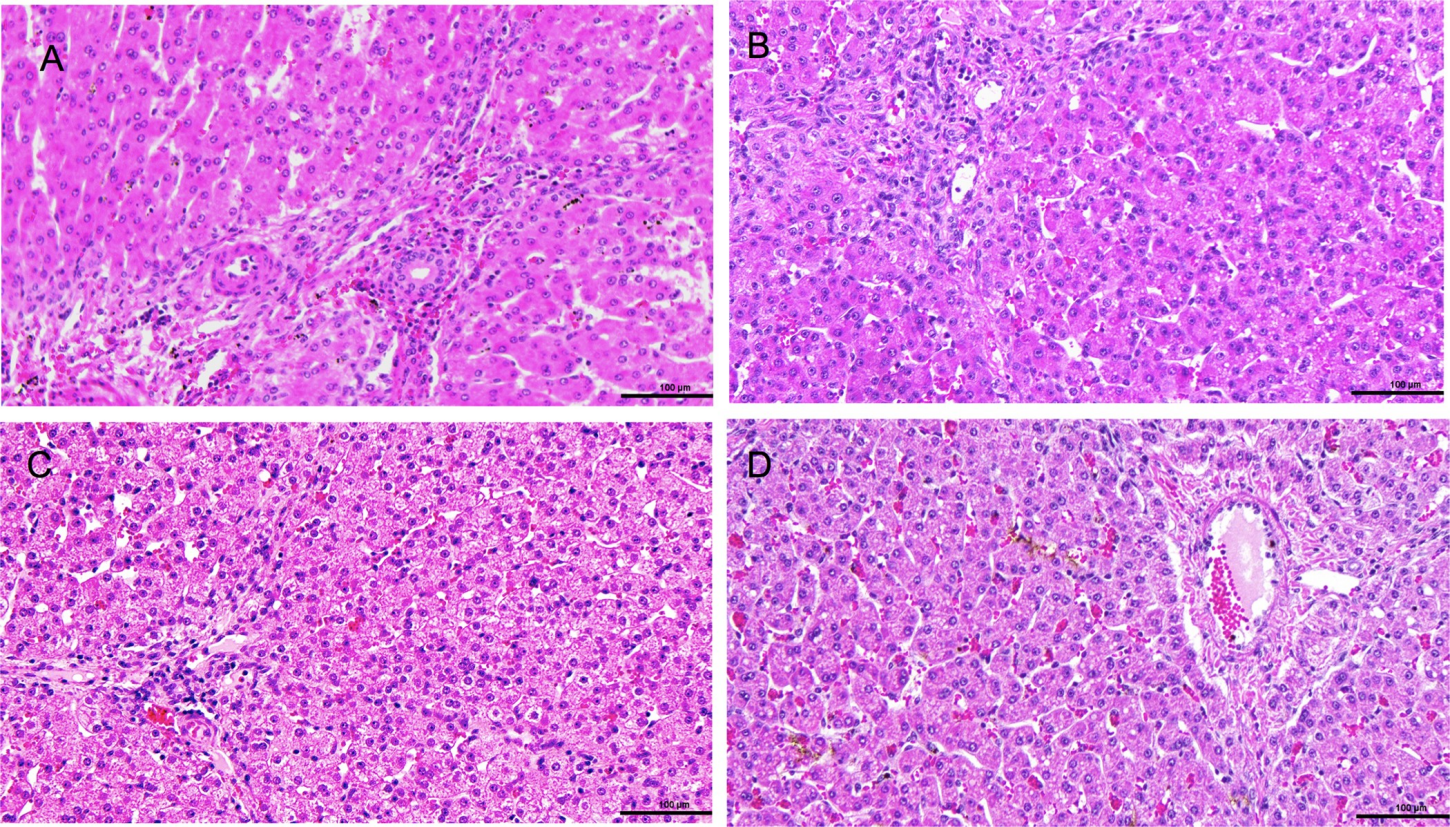
Representative histologic sections of liver parenchyma, stained with hematoxylin and eosin, taken at termination of 48 hours of perfusion. (A) Representative section of tissue from a liver in the intervention group. (B) Representative section of tissue from a liver in the intervention group. (C) Representative section of tissue from a liver in the control group. (D) Representative tissue from a liver in the control group.

Comparison of end of perfusion liver histology demonstrated evidence of hepatocyte injury in all liver biopsies, including hemorrhage, necrosis and sinusoidal dilatation. The severity however, was not different between groups (p>0.99) **(Fig 5).**

## Discussion

Liver *ex situ* NMP has previously demonstrated the potential to resuscitate donor livers that would otherwise be discarded from the transplant process [1]. Functional evaluation of such livers is necessary prior to implantation, in order to minimize the possibility of the catastophic outcome of primary non-function (PNF). *Ex situ* NMP offers a potentially ideal temperature modality, as it allows for viability assessment occur in the setting of physiologic metabolism.

Transaminase levels are ubiquitously used as a marker of graft hepatocellular injury, and indeed, as such have been used as an endpoint in most clinical liver *ex situ* studies [1, 7–9]. Further, transaminases in liver procurement flush solution and in machine perfusate have been shown to correlate with post-transplant graft function [4]. In previous studies, machine perfusate transaminase levels would be reported, but to-date, not one group has elucidated the kinetics of how these enzymes are potentially cleared in the context of an isolated, perfused liver circuit. The goal of the present study was to clarify this as an important means to interpret *ex situ* quantification and kinetics of transaminase degradation.

In this study, we found that liver transaminases increased in the control group at a slow rate, as previously described [12]. In the treatment group transaminase levels elevated promptly as anticipated at the intervention time-point, and then slowly decrease after. It is possible that a degree of hepatocellular injury resulting from the intervention could also have contributed to the observed enzyme elevation. The *in vivo* natural half-live of AST is 17 ± 5 hours, and the half life of ALT is 47 ± 10 hours, as reported previously [24]. Since the rate of transaminase decrease in the treatment group did not correspond to these half-lives, we surmise that the decrease in enzyme levels in the treatment group is in part the result of endogenous breakdown of these enzymes in the liver. It has been previously shown that transaminases are taken up by sinusoidal cells and cleared by Küpffer cells in the liver [25, 26]. The transaminase levels in the treatment group never decreased to the level of the control group, and we speculate that at a certain point, enzymatic transaminase catabolism reaches a steady state, with enzyme degradation equivalent to that of release from damaged cells. In this scenario, it is likely that at the end of the perfusion duration, some of the exogenous transaminases in the intervention group remained in the circulating perfusate. An unknown possibility whether the observed decrease in transaminase levels was a result of enzyme binding to components of the perfusion circuit. We cannot fully predict the outcome if these perfusions had been allowed to proceed for a longer duration.

LDH is a well-known marker of cellular injury, and has been suggested as an indicator of the quality of a liver graft, and ultimately its transplant potential [27]. In the control group, LHD increased and then reached a plateau, as previously demonstrated [27]. In the experimental group, LDH elevated significantly at the intervention time point, likely from an exogenous bolus of LDH present in the transaminase supernatant. Again, cannot say with certainty that any degree of hepatocellular injury resulting directly from the intervention did not contribute to the observed enzyme elevation.

Machine perfusate lactate levels are consistently reported as one of the most important markers of graft injury during perfusion. Indeed, any group that has suggested a composite series of viability markers for *ex situ* graft assessment has invariably included decreasing lactate as a dominant variable [4, 6, 16, 28]. Recently, it has been suggested that perhaps it is not just the absolute decrease of lactate levels, but the fall in lactate per weight of liver tissue, which may be of most utility. This is in view of the fact that in a recent clinical NMP series livers that had cleared lactate still went on to develop PNF [4].

In this study, all livers had a very minimal rise in lactate, ostensibly indicating minimal liver damage, in keeping with previously reported longer-term perfusion experiments [12, 21, 29]. Both control and treatment groups demonstrated similar lactate curves, indicating that the intervention did not compromise liver perfusion or lead to significant functional injury. Although perfusate lactate level is commonly used as a marker of liver damage, it is reported to be cleared by zone 1 of the hepatic lobule [30]. This zone is closest to the portal venules and arterioles, and as such is well perfused and oxygenated. Lactate levels would therefore likely only rise if a pan-lobular injury was present or if an entire liver segment was not perfused, as in the case of a damaged accessory artery. Nevertheless, lactate currently continues to be used by most groups in assessing *ex situ* viability, and indeed, we have discarded livers based on poor lactate clearance.

Data surrounding oxygen consumption by liver grafts undergoing *ex situ* perfusion is conflicting, with different groups reporting higher consumption associated with poor liver function, and others the converse. In the present study both control and treatment groups exhibited similar oxygen consumption curves, likely indicating similar liver metabolic demands and activity.

Vascular resistance has previously been described as a possible marker for graft viability, with the suggestion that a higher PVR was correlated to a graft with poorer function [3]. In this study, we found that HAR did not change significantly in either the intervention group or in the control group. PVR increased at the intervention time point in the treatment group, decreased to the level of controls, and then increased again from approximately 24 hours of perfusion. This was in contrast to the control livers, which demonstrated a consistently level PVR. This may possibly be explained in part by the fact that the concentrated transaminase solution, which was quite viscous in consistency and was injected into the portal vein at the intervention time-point, may have embolized distant venules within the liver parenchyma. Nevertheless, PVR did not rise to a level that would be considered deleterious to a graft, at least from previous reports [3].

To-date, bile production during *ex situ* liver perfusion was considered to be a potential surrogate marker of liver viability, with multiple groups suggesting this utility [3, 6, 16, 28, 31]. In theory, production of bile requires the integrity of multiple cellular and metabolic components, and as such would rationally implicate a well-functioning graft. This seemed to hold true in experimental large mammal *ex situ* experiments, with higher bile output correlating with improved post-transplant function. In clinical series however, this was not necessarily the case, with some livers that had produced bile proceeding to develop PNF post-transplant, and others that had produced no bile functioning well [4]. Recently, Matton *et al* reported that the absolute volume of bile may not be as critical as the bile quality, with recent findings demonstrating bile pH, glucose, and bicarbonate levels to correlate with ischemic cholangiopathy [32]. We observed that the total bile output in the treatment group was significantly higher than in the controls. This is not readily explainable as the formation and secretion of bile is a complex process dependent on various hormonal, electrolyte, neural, and humoral factors [33]. It had been previously shown that after 10 hours of experimental *ex situ* perfusion, bile production would slow down if the perfusate was not supplemented with exogenous bile salts. An infusion of taurocholate would maintain bile production at physiological levels (8 mL/h +/_ 0.75) throughout a 20 hour perfusion [34]. At the time of this study, we did not have access to commercial grade bile salts, and so did not supplement our perfusions. Despite this, all livers steadily produced bile, albeit not at the suggested physiological levels, likely due to a paucity of bile salts.

The presence of hepatocyte injury at the end of our perfusions, evident on histology, is likely due to progressive, cumulative on-circuit graft damage. This may be a result of the formation of reactive oxygen species, bile salt depletion, progressive mechanical perfusion injury, or damage as a direct result of the performed intervention.

There are several limitations to our study. The highly concentrated transaminase supernatant used to elevate circuit transaminase levels contains many cytoplasmic constituents and cell fragments, and we ultimately do not know how these interacted with each other and the metabolism of the livers during NMP. Consideration was given to using the tissue concentrates from other processed solid abdominal organs (kidney, spleen) in additional control perfusions, however, all tissue homogenates demonstrated elevated transaminases and LDH, and therefore would likely not contribute further clarity to the aim of the study. By not performing transplant experiments, we cannot ultimately predict how these livers would have functioned *in vivo*, or the resultant post-transplant biochemistry. However we clearly acknowledge that the perturbations observed in the perfusate-tainted tissue homogenate studies could be influenced by factors other than transaminase production and clearance, and our attempts to control for this using tissue homogenates other than the liver as control samples was unable to clarify this as these tissue too expressed high levels of transaminase.

Herein we have demonstrated that concentrated high levels of exogenous transaminases injected into a NMP ex situ circuit during perfusion of a healthy porcine liver are cleared from the perfusate, indicating preserved liver metabolism of transaminases. The functional status of the livers was not evidently affected by the increase in transaminase levels, as evidenced by similar lactate levels, and oxygen consumption between groups. Clearance of endogenous or exogenous transaminases during NMP may be a graft tolerance test that indicates good graft function and viability, with decreasing levels indicating good graft function.

## Supporting information

S1 File Experimental dataset. (XLSX)

## References

[1] Nasralla D, Coussios CC, Mergental H, Akhtar MZ, Butler AJ, Ceresa CDL, et al A randomized trial of normothermic preservation in liver transplantation. Nature. 2018 10.1038/s41586-018-0047-9 . 29670285

[2] Guarrera JV, Henry SD, Samstein B, Odeh-Ramadan R, Kinkhabwala M, Goldstein MJ, et al Hypothermic machine preservation in human liver transplantation: the first clinical series. Am J Transplant. 2010;10(2):372–81. 10.1111/j.1600-6143.2009.02932.x . 19958323

[3] Brockmann J, Reddy S, Coussios C, Pigott D, Guirriero D, Hughes D, et al Normothermic perfusion: a new paradigm for organ preservation. Ann Surg. 2009;250(1):1–6. 10.1097/SLA.0b013e3181a63c10 . 19561463

[4] Watson CJE, Kosmoliaptsis V, Pley C, Randle L, Fear C, Crick K, et al Observations on the ex situ perfusion of livers for transplantation. Am J Transplant. 2018;18(8):2005–20. 10.1111/ajt.14687 29419931 PMC6099221

[5] Watson CJE, Kosmoliaptsis V, Randle LV, Gimson AE, Brais R, Klinck JR, et al Normothermic Perfusion in the Assessment and Preservation of Declined Livers Before Transplantation: Hyperoxia and Vasoplegia-Important Lessons From the First 12 Cases. Transplantation. 2017;101(5):1084–98. 10.1097/TP.0000000000001661 . 28437389 PMC5642347

[6] Mergental H, Perera MT, Laing RW, Muiesan P, Isaac JR, Smith A, et al Transplantation of Declined Liver Allografts Following Normothermic Ex-Situ Evaluation. Am J Transplant. 2016;16(11):3235–45. 10.1111/ajt.13875 . 27192971

[7] Bral M, Gala-Lopez B, Bigam D, Kneteman N, Malcolm A, Livingstone S, et al Preliminary Single-Center Canadian Experience of Human Normothermic Ex Vivo Liver Perfusion: Results of a Clinical Trial. Am J Transplant. 2016 10.1111/ajt.14049 . 27639262

[8] Selzner M, Goldaracena N, Echeverri J, Kaths JM, Linares I, Selzner N, et al Normothermic ex vivo liver perfusion using steen solution as perfusate for human liver transplantation: First North American results. Liver Transpl. 2016;22(11):1501–8. 10.1002/lt.24499 . 27339754

[9] Ravikumar R, Jassem W, Mergental H, Heaton N, Mirza D, Perera MT, et al Liver transplantation after ex vivo normothermic machine preservation: a Phase 1 (first-in-man) clinical trial. Am J Transplant. 2016 10.1111/ajt.13708 . 26752191

[10] Watson CJ, Randle LV, Kosmoliaptsis V, Gibbs P, Allison M, Butler AJ. 26-hour Storage of a Declined Liver Before Successful Transplantation Using Ex Vivo Normothermic Perfusion. Ann Surg. 2016 10.1097/SLA.0000000000001834 . 27295096

[11] Perera T, Mergental H, Stephenson B, Roll GR, Cilliers H, Liang R, et al First human liver transplantation using a marginal allograft resuscitated by normothermic machine perfusion. Liver Transpl. 2016;22(1):120–4. 10.1002/lt.24369 . 26566737

[12] Vogel T, Brockmann JG, Pigott D, Neil DAH, Muthusamy ASR, Coussios CC, et al Successful transplantation of porcine liver grafts following 48-hour normothermic preservation. PLoS One. 2017;12(11):e0188494 10.1371/journal.pone.0188494 29176869 PMC5703476

[13] Vogel T, Brockmann JG, Quaglia A, Morovat A, Jassem W, Heaton ND, et al 24-Hour Normothermic Machine Perfusion of Discarded Human Liver Grafts. Liver Transpl. 2016 10.1002/lt.24672 . 27809409

[14] Banan B, Chung H, Xiao Z, Tarabishy Y, Jia J, Manning P, et al Normothermic extracorporeal liver perfusion for donation after cardiac death (DCD) livers. Surgery. 2015;158(6):1642–50. 10.1016/j.surg.2015.07.016 . 26294088

[15] Nassar A, Liu Q, Farias K, D'Amico G, Tom C, Grady P, et al Ex vivo normothermic machine perfusion is safe, simple, and reliable: results from a large animal model. Surg Innov. 2015;22(1):61–9. 10.1177/1553350614528383 . 24694840

[16] op den Dries S, Karimian N, Sutton ME, Westerkamp AC, Nijsten MW, Gouw AS, et al Ex vivo normothermic machine perfusion and viability testing of discarded human donor livers. Am J Transplant. 2013;13(5):1327–35. 10.1111/ajt.12187 . 23463950

[17] Boehnert MU, Yeung JC, Bazerbachi F, Knaak JM, Selzner N, McGilvray ID, et al Normothermic acellular ex vivo liver perfusion reduces liver and bile duct injury of pig livers retrieved after cardiac death. Am J Transplant. 2013;13(6):1441–9. 10.1111/ajt.12224 . 23668775

[18] Xu H, Berendsen T, Kim K, Soto-Gutierrez A, Bertheium F, Yarmush ML, et al Excorporeal normothermic machine perfusion resuscitates pig DCD livers with extended warm ischemia. J Surg Res. 2012;173(2):e83–8. 10.1016/j.jss.2011.09.057 22099594 PMC3682784

[19] Fondevila C, Hessheimer AJ, Maathuis MH, Munoz J, Taura P, Calatayud D, et al Superior preservation of DCD livers with continuous normothermic perfusion. Ann Surg. 2011;254(6):1000–7. 10.1097/SLA.0b013e31822b8b2f . 21862925

[20] Reddy SP, Bhattacharjya S, Maniakin N, Greenwood J, Guerreiro D, Hughes D, et al Preservation of porcine non-heart-beating donor livers by sequential cold storage and warm perfusion. Transplantation. 2004;77(9):1328–32. . 15167586 10.1097/01.tp.0000119206.63326.56

[21] Butler AJ, Rees MA, Wight DG, Casey ND, Alexander G, White DJ, et al Successful extracorporeal porcine liver perfusion for 72 hr. Transplantation. 2002;73(8):1212–8. . 11981411 10.1097/00007890-200204270-00005

[22] Schon MR, Kollmar O, Wolf S, Schrem H, Matthes M, Akkoc N, et al Liver transplantation after organ preservation with normothermic extracorporeal perfusion. Ann Surg. 2001;233(1):114–23. 11141233 10.1097/00000658-200101000-00017 PMC1421174

[23] Bral M, Gala-Lopez B, Thiesen A, Hatami S, Bigam DL, Freed DM, et al Determination of Minimal Hemoglobin Level Necessary for Normothermic Porcine Ex Situ Liver Perfusion. Transplantation. 2018;102(8):1284–92. 10.1097/TP.0000000000002272 . 29757899

[24] Kim WR, Flamm SL, Di Bisceglie AM, Bodenheimer HC, Public Policy Committee of the American Association for the Study of Liver D. Serum activity of alanine aminotransferase (ALT) as an indicator of health and disease. Hepatology. 2008;47(4):1363–70. 10.1002/hep.22109 . 18366115

[25] Radi ZA, Koza-Taylor PH, Bell RR, Obert LA, Runnels HA, Beebe JS, et al Increased serum enzyme levels associated with kupffer cell reduction with no signs of hepatic or skeletal muscle injury. Am J Pathol. 2011;179(1):240–7. 10.1016/j.ajpath.2011.03.029 21703406 PMC3123844

[26] Kamimoto Y, Horiuchi S, Tanase S, Morino Y. Plasma clearance of intravenously injected aspartate aminotransferase isozymes: evidence for preferential uptake by sinusoidal liver cells. Hepatology. 1985;5(3):367–75. . 3997068 10.1002/hep.1840050305

[27] Monbaliu D, Liu Q, Libbrecht L, De Vos R, Vekemans K, Debbaut C, et al Preserving the morphology and evaluating the quality of liver grafts by hypothermic machine perfusion: a proof-of-concept study using discarded human livers. Liver Transpl. 2012;18(12):1495–507. 10.1002/lt.23550 . 22987314

[28] Mergental H, Stephenson BTF, Laing RW, Kirkham AJ, Neil DAH, Wallace LL, et al Development of Clinical Criteria for Functional Assessment to Predict Primary Nonfunction of High-Risk Livers Using Normothermic Machine Perfusion. Liver Transpl. 2018;24(10):1453–69. 10.1002/lt.25291 . 30359490 PMC6659387

[29] Liu Q, Nassar A, Buccini L, Grady P, Soliman B, Hassan A, et al Ex situ 86-hour liver perfusion: Pushing the boundary of organ preservation. Liver Transpl. 2018;24(4):557–61. 10.1002/lt.25007 . 29316185

[30] Watson CJE, Jochmans I. From "Gut Feeling" to Objectivity: Machine Preservation of the Liver as a Tool to Assess Organ Viability. Curr Transplant Rep. 2018;5(1):72–81. 10.1007/s40472-018-0178-9 29564205 PMC5843692

[31] Sutton ME, op den Dries S, Karimian N, Weeder PD, de Boer MT, Wiersema-Buist J, et al Criteria for viability assessment of discarded human donor livers during ex vivo normothermic machine perfusion. PLoS One. 2014;9(11):e110642 10.1371/journal.pone.0110642 25369327 PMC4219693

[32] Matton APM, de Vries Y, Burlage LC, van Rijn R, Fujiyoshi M, de Meijer VE, et al Biliary Bicarbonate, pH and Glucose Are Suitable Biomarkers of Biliary Viability During Ex Situ Normothermic Machine Perfusion of Human Donor Livers. Transplantation. 2018 10.1097/TP.0000000000002500 . 30395120 PMC6613725

[33] Boyer JL. Bile formation and secretion. Compr Physiol. 2013;3(3):1035–78. 10.1002/cphy.c120027 23897680 PMC4091928

[34] Imber CJ, St Peter SD, de Cenarruzabeitia IL, Lemonde H, Rees M, Butler A, et al Optimisation of bile production during normothermic preservation of porcine livers. Am J Transplant. 2002;2(7):593–9. . 12201359 10.1034/j.1600-6143.2002.20703.x

